# Epstein–Barr virus nuclear antigen (EBNA) 3A induces the expression of and interacts with a subset of chaperones and co-chaperones

**DOI:** 10.1099/vir.0.83414-0

**Published:** 2008-04

**Authors:** Paul Young, Emma Anderton, Kostas Paschos, Rob White, Martin J. Allday

**Affiliations:** Department of Virology, Imperial College London, St Mary's Campus, Norfolk Place, London W2 1PG, UK

## Abstract

Viral nuclear oncoproteins EBNA3A and EBNA3C are essential for the efficient immortalization of B cells by Epstein–Barr virus (EBV) *in vitro* and it is assumed that they play an essential role in viral persistence in the human host. In order to identify cellular genes regulated by EBNA3A expression, cDNA encoding EBNA3A was incorporated into a recombinant adenoviral vector. Microarray analysis of human diploid fibroblasts infected with either adenovirus EBNA3A or an empty control adenovirus consistently showed an EBNA3A-specific induction of mRNA corresponding to the chaperones Hsp70 and Hsp70B/B′ and co-chaperones Bag3 and DNAJA1/Hsp40. Analysis of infected fibroblasts by real-time quantitative RT-PCR and Western blotting confirmed that EBNA3A, but not EBNA3C, induced expression of Hsp70, Hsp70B/B′, Bag3 and DNAJA1/Hsp40. This was also confirmed in a stable, inducible expression system. EBNA3A activated transcription from the Hsp70B promoter, but not multimerized heat-shock elements in transient transfection assays, consistent with specific chaperone and co-chaperone upregulation. Co-immunoprecipitation experiments suggest that EBNA3A can form a complex with the chaperone/co-chaperone proteins in both adenovirus-infected cells and EBV-immortalized lymphoblastoid cell lines. Consistent with this, induction of EBNA3A resulted in redistribution of Hsp70 from the cytoplasm to the nucleus. EBNA3A therefore specifically induces (and then interacts with) all of the factors necessary for an active Hsp70 chaperone complex.

## INTRODUCTION

EBNAs 3A, 3B and 3C are three related Epstein–Barr virus (EBV) latency-associated proteins that are expressed in lymphoblastoid cell lines (LCLs) produced by *in vitro* infection of primary B cells with EBV. Genetic analysis has revealed that EBNA3A and EBNA3C are two of six latency-associated genes that are essential for the creation of LCLs (Maruo *et al.*, 2003; Tomkinson & Kieff, 1992; Tomkinson *et al.*, 1993). EBNAs 3A, 3B and 3C are a related family of nuclear proteins that have modest amino acid sequence similarity; their genes have a similar structure (a short 5′ exon followed by a long 3′ exon), are arranged tandemly in the genome and are transcribed from the common promoter (Cp) (reviewed by Bornkamm & Hammerschmidt, 2001). The EBNA3 proteins are unable to bind to DNA directly, but all three bind to a cellular DNA-binding factor, CBF1/RBP-J*κ* (Robertson *et al.*, 1996). EBNA3A and EBNA3C repress transcription of the viral promoters TP1 and Cp through interaction with RBP-J*κ* and can inhibit EBNA2-mediated activation of these promoters (Bain *et al.*, 1996; Cludts & Farrell, 1998; Le Roux *et al.*, 1994). In addition, EBNA3A and EBNA3C have been shown to repress transcription when targeted artificially to a promoter through a Gal4 DNA-binding domain (Bain *et al.*, 1996; Cludts & Farrell, 1998). Repression by EBNA3A and EBNA3C is probably associated with the ability of both proteins to recruit repression complexes, which can alter histone acetylation and/or methylation (Hickabottom *et al.*, 2002; Knight *et al.*, 2003; Radkov *et al.*, 1999; Touitou *et al.*, 2001). Both EBNA3A and EBNA3C bind to CtBP1 (E1A C-terminal-binding protein), a highly conserved co-repressor that is associated with gene silencing (Chinnadurai, 2002; Hickabottom *et al.*, 2002; Touitou *et al.*, 2001).

All of the available evidence suggests that the EBNA3 proteins should regulate transcription, and gene regulation mediated by the EBNA3 proteins is unlikely to be limited to viral promoters. As EBV is associated with B-cell growth transformation and several lymphomas (Rickinson & Kieff, 2007), it has been predicted that EBNA3A and EBNA3C also modulate cellular gene targets involved in cell survival or proliferation. However, identification and characterization of bona fide cellular targets of EBNA3A have, to date, been largely unsuccessful.

Here, we show that EBNA3A can upregulate a specific subset of cellular gene targets, including the chaperones Hsp70 and 70B/B′ and the co-chaperones Hsp40 and Bag3. This upregulation was initially shown by microarray analysis using recombinant adenoviruses expressing EBNA3A, but was confirmed by real-time quantitative (q) RT-PCR and Western blot analyses. Importantly, EBNA3A also interacts with the same subset of chaperones and co-chaperones at physiological levels in LCLs. Consistent with the observed upregulation of Hsp70B mRNA, EBNA3A has been found to activate transcription of the Hsp70B promoter and to co-localize in the nucleus with Hsp70 protein.

## METHODS

### Cell culture.

DG75 cells (a Burkitt's lymphoma-derived cell line) and LCLs were grown in suspension with RPMI 1640 medium (Invitrogen). IMR-90 cells (human fetal lung fibroblasts) were maintained in Eagle's minimum essential medium (Sigma). The HEK-293A cell line is an immortalized line of primary human embryonic kidney cells transformed by sheared human adenovirus type 5 (Ad5) DNA. The E1A and E1B region of the adenoviral genome, which complements *in* *trans* the deletion of the E1 region in the recombinant adenovirus, is expressed in 293A cells. The p53-negative human lung carcinoma cell line H1299 and 293A cells were cultured in Dulbecco's modified Eagle's medium (Invitrogen). All media were supplemented with 10 % fetal calf serum, penicillin/streptomycin (100 U ml^−1^) and glutamine (2 mM).

### Adenovirus construction.

Adenovirus production utilized the pAdEasy-1 system of recombinant adenoviruses (He *et al.*, 1998; Zeng *et al.*, 2001). The system uses homologous recombination in *recA^+^* *Escherichia coli* strain BJ5183 to introduce the gene of interest into the adenovirus background. Initial cloning is performed into a shuttle vector (p-Shuttle CMV) that has two regions of homology to the adenovirus backbone plasmid (pAdEasy-1) and is devoid of E1 and E3. EBNA3A and the EBNA3A–CtBP binding mutant were cloned from pcDNA3-HA-EBNA3A and pcDNA3-HA-EBNA3A–CtBP, respectively [as detailed by Hickabottom *et al.* (2002), with the CtBP-binding mutant described previously as the 2× mutant]. EBNA3C was cloned from a pcDNA3-EBNA3C vector via a pBlueScript II SK+ cloning intermediate. Recombinant adenoviral vectors were propagated in 293A packaging cells and scaled up before CsCl purification and determination of the virus titre (for more information, please refer to the Supplementary Methods, available in JGV Online).

### Microarray analysis.

IMR-90 cells were infected (m.o.i. of 25) with either adenovirus expressing EBNA3A or an empty adenovirus, which served as a negative control. Virus infection was for 6 h before the virus was removed and replaced with supplemented medium. Microarrays compared adenovirus-EBNA3A with an empty control. Total cell RNA was extracted 24 h post-infection with an RNeasy Mega kit (Qiagen). Samples of the RNA were analysed by RNase-protection assay for GAPDH (glyceraldehyde-3-phosphate dehydrogenase) content to ensure that the RNA was intact. RNA aliquots (50 μg) from control and test cells were then labelled in reverse transcription (RT) reactions with dCTP–Cy3 and dCTP–Cy5. Control RNA labelled with Cy3 was mixed with test RNA labelled with Cy5, and vice versa. Both mixes were used in overnight hybridization reactions with Ludwig/Sanger/ICRF Consortium microarray chips (Hver 2.1.1). The chips contained 15 000 sequence-verified probes (see http://www.sanger.ac.uk/Projects/Microarrays/ for more details of the protocols). Labelling test samples with both Cy3 and Cy5 enabled us to minimize the variation within the experiment caused by differential labelling with two different dyes. The slides were washed and then scanned by using a GSI Lumonics ScanArray 4000 and analysed by using QuantArray software. Three independent experiments, with six chips per independent experiment (incorporating a dye swap), were collated by using GeneSpring analysis software (Silicon Genetics/Agilent). Data were Lowess-normalized.

### Real-time qRT-PCR analysis.

Primers for Bag3, Hsp70B/B′ and Hsp40 (DNAJA1) were obtained from SuperArray (Tebu-Bio). The primers for Hsp70B/B′ were not specific for either Hsp70B or B′, due to the highly conserved nature of the gene sequence and lack of introns (Leung *et al.*, 1990). A Qiagen SYBR Green RT-PCR kit with HotStarTaq DNA polymerase was used with an ABI Prism 7700 (Applied Biosystems). Real-time qRT-PCR conditions were RT for 30 min at 50 °C, using 250 ng RNA; melting for 15 min at 95 °C; and 30 cycles of three-step PCR, including melting for 30 s at 95 °C, annealing for 30 s at 55 °C and elongation for 30 s at 72 °C. Melting-curve analysis was performed to maintain single products and products were verified by agarose-gel electrophoresis. GAPDH was run as an endogenous control and all samples were normalized to GAPDH levels before comparison of fold induction.

### Immunoblotting and antibodies.

RIPA cell lysates were prepared and Western blotting was performed essentially as described previously (Touitou *et al.*, 2001). Membranes were blocked with 5 % milk powder in PBS/0.05 % Tween 20. After blocking, membranes were probed with the following antibodies: anti-Hsp70B′ (SPA756), anti-Hsp40 (SPA400), anti-Hsp90*α* (SPA771), anti-Bag1 (AAM400) and anti-Hsp70 (SPA810) (all from Stressgen). Anti-EBNA3C (A10) was a kind gift from Martin Rowe (Division of Cancer Studies, University of Birmingham Medical School, Birmingham, UK), anti-Bag3 was a kind gift from Alessandra Tosco (Università degli Studi di Salerno, Salerno, Italy) and polyclonal sheep anti-EBNA3A was purchased from ExAlpha.

### Immunoprecipitation (IP).

IP was performed as described previously (Hickabottom *et al.*, 2002; Radkov *et al.*, 1997; Touitou *et al.*, 2005). Two hundred micrograms of protein in a final volume of 200 μl, diluted in IP lysis buffer, was used per IP. Protein G–Sepharose beads were used to collect the immunoprecipitated complexes and the beads were washed five times in IP lysis buffer before SDS-PAGE for Western blot analysis.

### B-cell isolation, culture and infection with EBV.

Primary B cells were isolated from mixed-donor platelet-depleted buffy-coat residues by CD19 positive selection, as described previously (O';Nions & Allday, 2004; Spender *et al.*, 1999). Flow-cytometric analysis was performed by using fluorescein isothiocyanate-conjugated anti-CD20 mAbs (Dako) to monitor cell purity. Purified B cells were found to be between 80 and 90 % pure. CD40 stimulation and infection with EBV have been described previously (O';Nions & Allday, 2004; Spender *et al.*, 1999).

### Inducible EBNA3A.

The vectors for inducible expression of EBNA3A and EBNA3A–CtBP mutant were made by cloning the haemagglutinin (HA)-tagged versions of the wild-type gene and mutant into expression vector pRTS1 [as described by Bornkamm *et al.* (2005), but with a puromycin-resistance gene instead of hygromycin B] in place of the luciferase gene. The resultant plasmids have an inducible, bidirectional promoter driving the expression of EBNA3A or EBNA3A–CtBP mutant in one direction and enhanced green fluorescent protein (eGFP) in the other. They also contain the EBV gene EBNA1 and the EBV episomal origin of replication (oriP) and are maintained episomally.

These plasmids and the parental RTS-1 (2 μg) were transfected into H1299 cells with Lipofectamine 2000 as per the manufacturer's instructions (Invitrogen). Forty-eight hours after transfection, 1 μg puromycin ml^−1^ (Calbiochem) was added. After 10 days, surviving cells grown out were transferred into T25 flasks. Cells were induced with 1 μg doxycycline ml^−1^ (Sigma) and eGFP was seen under a microscope in 100 % of cells after 24 h, indicating the presence of the plasmid in all cells. Expression of EBNA3A and EBNA3A–CtBP binding mutant was verified by Western blot.

### Hsp70 immunofluorescence.

H1299, H1299-RTS1 and H1299-3A cells were seeded onto coverslips and incubated in the presence or absence of 1 μg doxycycline ml^−1^ for 24 h. Cells were fixed with 4 % paraformaldehyde in PBS and permeabilized with 0.2 % Triton X-100. The coverslips were blocked in 1 % BSA/PBS and incubated for 1 h at 37 °C with mouse monoclonal Hsp70 (SPA-810, 1 : 50) or mouse monoclonal EBNA3A (a kind gift from Martin Rowe, used undiluted). After washing in PBS, the slides were incubated with tetramethylrhodamine isothiocyanate (TRITC)-conjugated goat anti-mouse secondary antibody (1 : 100; Sigma). Slides were washed, MOWIOL-mounted and visualized on a Zeiss LSM 510 META confocal microscope.

### Transient transfections and *β*-galactosidase and reporter assays.

DG75 cells were transfected for reporter assays by electroporation as described previously (Hickabottom *et al.*, 2002), typically with a total of 10 μg expression vector. IMR-90 cells were transfected by using the LID transfection system, consisting of Lipofectin reagent (Invitrogen), integrin-targeting peptide 6 (Hart *et al.*, 1998; White *et al.*, 2003) and DNA (for more information on transfection protocols and reporters, please refer to the Supplementary Methods, available in JGV Online).

Transfected DG75 cells were harvested and lysed in 100 μl 1× reporter lysis buffer (Promega) for 15 min. IMR-90 cells were lysed on the plate for 15 min in 150 μl of 1× cell lysis reagent (Promega). Twenty microlitres of cleared supernatant per transfection was used in the luciferase assay or in the *β*-galactosidase assay, as described previously (Hickabottom *et al.*, 2002). All luciferase values were normalized to *β*-galactosidase activity in the lysate.

## RESULTS

**Fig. 1. f1:**
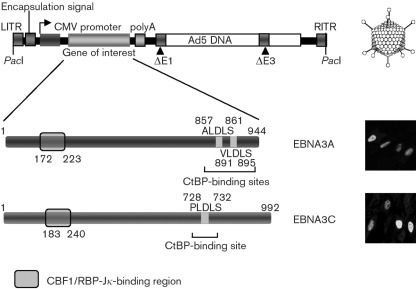
Schematic showing the pAdEasy-1 recombinant adenovirus backbone and the EBNA3A and EBNA3C adenoviruses used in this study. The CtBP-binding site(s) (A/V/PLDLS) and predicted CBF1/RBP-J*κ*-binding region are shown for EBNA3A and EBNA3C. Immunofluorescence images of infected IMR-90 cells (m.o.i. of 25, 24 h), using antibodies specific for EBNA3A or 3C, are shown in the bottom right panels. CMV, Cytomegalovirus; LITR, left internal repeat; RITR, right internal repeat.

Replication-defective adenoviruses (devoid of E1) expressing EBNA3A (Ad-3A) or EBNA3C (Ad-3C) are shown schematically in Fig. 1. A control adenovirus lacking an inserted gene (Ad-E) was also produced. EBNA3A expression was validated by immunofluorescence staining (>70 % cells were positive) and Western blotting (Figs 1 and 2d[Fig f2]).

### Microarray analysis shows that EBNA3A induces all of the factors thought to be necessary for an Hsp70 chaperone complex

**Fig. 2. f2:**
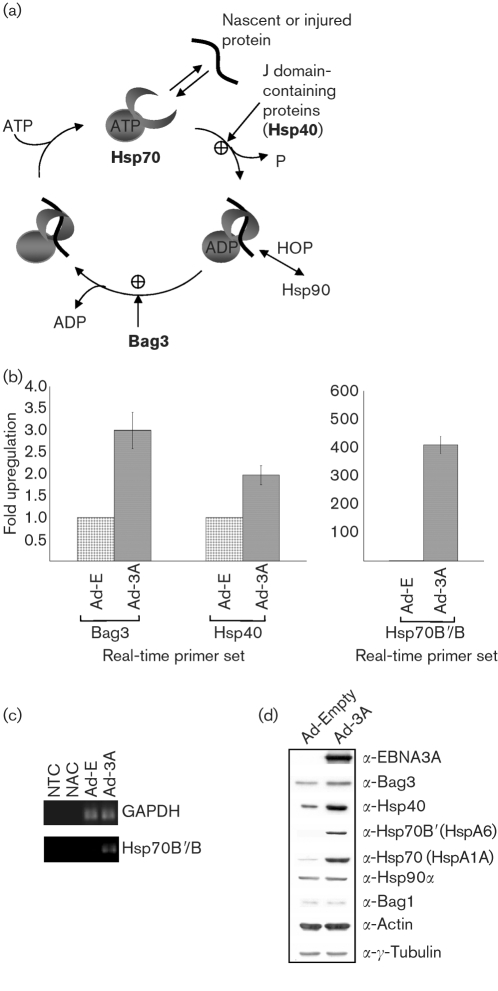
EBNA3A induces the co-chaperones necessary for the ATPase cycle of Hsp70. (a) The Hsp70 family is one of the most highly conserved gene families and consists of both constitutive and inducible forms. Hsp70 has a dual function and can either form a complex with a nascent or misfolded protein and chaperone them for refolding, or remove proteins by polyubiquitination and proteasome-mediated degradation (Bukau & Horwich, 1998; Esser *et al.*, 2004). Protein binding by Hsp70 is linked intrinsically to its ATP/ADP-binding status, modulated through the N-terminal ATPase domain (Greene *et al.*, 1995; McCarty *et al.*, 1995; Palleros *et al.*, 1993). Co-chaperones have been identified that can regulate the peptide-binding cycle of Hsp70, by either augmenting or inhibiting protein binding, or by targeting the chaperone to a specific subcellular compartment or protein (reviewed by Mayer, 2005). The co-chaperones Hsp40 and Bag3 (shown in bold) were shown to be upregulated by EBNA3A expression following adenovirus infection of IMR-90 cells. (b) Summary of real-time qRT-PCR analysis using all of the RNA samples from Ad-3A versus Ad-E microarray experiments. GAPDH was run as an endogenous control against which all samples were normalized. (c) End-point real-time qRT-PCR samples were resolved on an agarose gel, demonstrating little to no Hsp70B/B′ DNA product with Ad-E infection. This is consistent with Hsp70B/B′ being silent in most cells. NTC, No template control; NAC, no activation (no reverse transcriptase) control. (d) Western blot analysis of protein samples taken at the time of cell harvesting for the microarray experiments (Table 1). A representative series of blots from one set of protein samples is shown, probed with the antibodies indicated.

Microarray analysis was used to compare the infection of IMR-90 cells with Ad-3A or Ad-E. The data from microarray analysis, 24 h post-infection, showed that EBNA3A expression induced a specific subset of chaperones and co-chaperones, including Hsp70, Hsp70B/B′, Bag3 and Hsp40 (Table 1). This upregulation was particularly significant given the small number of genes induced in this system (*n*=10; see Supplementary Tables S1 and S2, available in JGV Online, for a full list). The specific subset of chaperones and co-chaperones induced by Ad-3A are all part of an Hsp70 ATPase cycle (Fig. 2a[Fig f2]). These results suggest a co-ordinated regulation of a specific pathway of the cell stress response by EBNA3A.

### Real-time qRT-PCR and Western blot analysis confirmed specific upregulation of Hsp70B/B′, Bag3 and Hsp40 by EBNA3A

Real-time qRT-PCR experiments were performed to validate the expression data obtained from the microarrays. Aliquots of the RNA generated for microarray experiments were combined with real-time qRT-PCR primers corresponding to Bag3, Hsp40(DNAJA1) and Hsp70B/B′ (these primers detect both Hsp70B and Hsp70B′ transcripts). Comparison of Ad-3A- with Ad-E-infected IMR-90 cells showed approximately 3-fold and 2-fold upregulation for Bag3 and Hsp40, respectively (Fig. 2b[Fig f2]). The observed induction of the two co-chaperones was consistent with the normalized microarray data (Table 1). Real-time qRT-PCR results for Hsp70B/B′, comparing Ad-3A and Ad-E, gave an approximately 400-fold upregulation. Hsp70B/B′ are basic, heat-inducible forms of Hsp70 and have no mRNA expression under normal physiological conditions. However, cellular stresses, such as heat shock, are known to induce Hsp70B′ mRNA (Leung *et al.*, 1990). The observed induction by EBNA3A was consistent with Hsp70B/B′ being in an activated or ‘on’ state with EBNA3A expression, but silent when the cells were infected with the control adenovirus. Agarose-gel analysis of the end products of a representative real-time qRT-PCR experiment showed little to no signal with Ad-E, but clear Hsp70B/B′ transcript signal with Ad-3A (Fig. 2c[Fig f2]).

To determine whether the increase in RNA was reflected in the accumulation of protein, Western blot analysis was performed on protein samples taken at the time of RNA harvesting for microarray analysis (Fig. 2d[Fig f2]). Representative protein samples showed that the levels of chaperones Hsp70 (HspA1A) and Hsp70B′ (HspA6) and of the co-chaperones Hsp40 and Bag3 were increased when Ad-3A-infected IMR-90 cells were compared with Ad-E-infected control cells. Importantly, Hsp70B′ was undetectable when IMR-90 cells were infected with Ad-E, but induced to a high level when Ad-3A was expressed. These results are consistent with the RNA data described above.

### EBNA3C does not induce chaperone or co-chaperone RNA or protein

**Fig. 3. f3:**
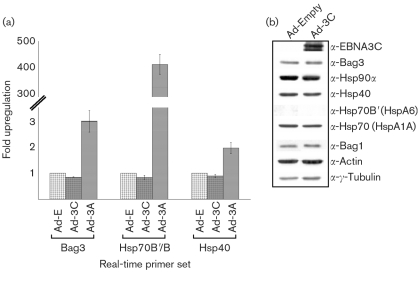
No increase in chaperones or co-chaperones was observed after infection with adenovirus EBNA3C. (a) Real-time qRT-PCR analysis with primers for Bag3, Hsp40 and Hsp70B/B′. No significant increase in Bag3, Hsp40 or Hsp70B/B′ was observed with Ad-3C (m.o.i. of 25, 24 h) compared with Ad-E control infection (Ad-3A infection results are included for comparison). GAPDH is run as an endogenous control to which all samples are normalized. (b) Western blot analysis of protein samples taken from IMR-90 cells infected with Ad-3C versus Ad-E control infection. No increase in chaperones or co-chaperones was seen with EBNA3C expression.

The specificity of the induction of chaperones and co-chaperones by EBNA3A was confirmed by comparison of the above-described results with the responses obtained when IMR-90 cells were infected with Ad-3C, which encodes the related protein EBNA3C. The results obtained with Ad-3C were comparable to those with Ad-E, i.e. failure to induce expression of Bag3, Hsp40 or Hsp70B/B′, as shown by real-time qRT-PCR analysis (Fig. 3a[Fig f3]). Western blot analysis of IMR-90 cells infected with Ad-3C compared with Ad-E-infected control cells confirmed that EBNA3C was expressed at a similar level to EBNA3A, but that it did not induce any of the chaperones or co-chaperones (Fig. 3b[Fig f3]).

### EBNA3A and a subset of chaperones and co-chaperones interact in cells

**Fig. 4. f4:**
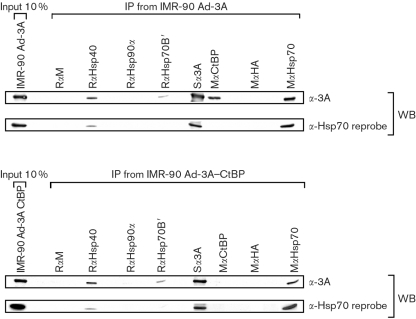
EBNA3A binds to Hsp70, Hsp70B′ and the co-chaperone Hsp40 in adenovirus-infected IMR-90 cells. IP from adenovirus-infected IMR-90 cells (m.o.i. of 25, 24 h) using Ad-3A, Ad-3A–CtBP binding mutant and endogenous chaperones and co-chaperones. EBNA3A or EBNA3A–CtBP were co-precipitated with an anti-Hsp70, anti-Hsp70B′ or anti-Hsp40 antibody and detected by Western blot analysis with an anti-EBNA3A antibody. No binding to Hsp90*α* was observed. Two hundred micrograms of cell lysate was used per IP and all antibodies were used at a 1 : 100 dilution, except anti-CtBP, which was used at a 1 : 200 dilution. Hsp70 antibody was subsequently used to reprobe the membrane and is shown below the respective EBNA3A Western blots. R*α*M, Rabbit anti-mouse antibody; R*α*Hsp40, rabbit anti-Hsp40; R*α*Hsp90*α*, rabbit anti-Hsp90*α*; R*α*Hsp70B′, rabbit anti-Hsp70B′; S*α*3A, sheep anti-EBNA3A; R*α*CtBP, rabbit anti-CtBP; M*α*HA, mouse anti-haemagglutinin; M*α*Hsp70, mouse anti-Hsp70.

Experiments were performed to determine whether EBNA3A and the upregulated chaperones and co-chaperones interact in IMR-90 cells. In these experiments, the double EBNA3A–CtBP binding mutant [as described by Hickabottom *et al.* (2002)] was included, as CtBP binding is important in gene regulation and protein–protein interactions. Protein extracts from Ad-3A- or Ad-3A–CtBP-infected IMR-90 cells were subjected to IP with antibodies directed against Hsp70, Hsp70B′, Hsp40 or Hsp90*α*, or an irrelevant antibody control, and subsequently Western-blotted and probed with an anti-EBNA3A polyclonal antibody. Representative results shown in Fig. 4 demonstrate that both EBNA3A and the EBNA3A–CtBP binding mutant co-precipitate with Hsp70, Hsp70B′ and Hsp40, but not with Hsp90*α* or the control antibody. As expected, the Ad-3A–CtBP binding mutant failed to co-immunoprecipitate with endogenous CtBP. Reprobing the membrane with an antibody specific for Hsp70 (HspA1A) revealed the reverse co-IP of EBNA3A with Hsp70, using an anti-EBNA3A IP and anti-Hsp70 Western blot. Reprobing the filter further established that IP with anti-Hsp40 resulted in precipitation of both Hsp70 and EBNA3A, as shown by the representative Western blots. Hsp40 interaction with Hsp70 has been shown previously (Demand *et al.*, 1998), but the presence of EBNA3A with Hsp70 and Hsp40 suggests a chaperone–co-chaperone ternary complex. The interaction of Ad-3A (and the CtBP binding mutant) with chaperones and co-chaperones was in contrast to that of Ad-3C, which failed to co-immunoprecipitate the same endogenous chaperones and co-chaperones in IMR-90 cells (data not shown).

### Endogenous EBNA3A and chaperones/co-chaperones co-precipitate in LCLs, and Hsp70B′ is upregulated in these cells

**Fig. 5. f5:**
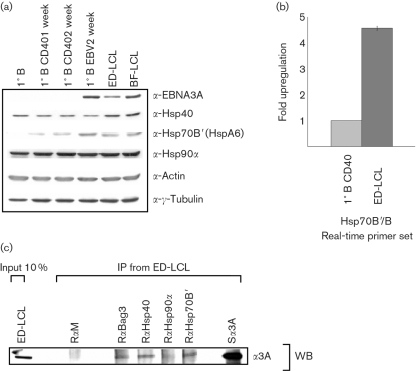
Interaction of Hsp70B′ and the co-chaperones Hsp40 and Bag3 with EBNA3A expressed in LCLs. (a) Western blot analysis of primary B cells, primary B cells stimulated with CD40L/IL-4, primary B cells infected with EBV and established LCLs (ED and BF) confirmed expression of chaperones and co-chaperones in LCLs. Hsp70B′ was expressed at a relatively high level in LCLs and primary B cells infected with EBV, but at a lower level in B cells stimulated with CD40L/IL-4, and was absent in non-stimulated resting primary B cells. (b) The increase in Hsp70B/B′ seen in LCLs compared with CD40L/IL-4-stimulated B cells was confirmed by real-time qRT-PCR analysis. (c) Co-IP of EBNA3A with chaperones and co-chaperones in LCLs. Two hundred micrograms of protein from ED-LCL cell line was immunoprecipitated with a 1 : 100 dilution of anti-Hsp antibodies and subsequently Western-blotted with an anti-EBNA3A antibody.

LCLs express the complete repertoire of EBV latent proteins at their physiologically appropriate levels. LCLs were initially Western-blotted to determine whether the chaperones and co-chaperones were expressed. Comparison of resting primary CD19^+^ B cells, primary B cells stimulated with CD40L/interleukin-4 (IL-4) for 1 and 2 weeks, similar B cells infected with EBV for 2 weeks and established LCLs (LCL-BF and LCL-ED) demonstrated the presence of endogenous chaperones and co-chaperones in all of these B cells. It was noted that Hsp70B′ was undetectable in primary resting B cells and was expressed at a low level in CD40L/IL-4-stimulated B cells, but expression was increased significantly in primary B cells infected with EBV and in established LCLs (Fig. 5a[Fig f5]). The upregulation of Hsp70B′ in EBV-infected primary B cells and LCLs was confirmed by real-time qRT-PCR analysis. The amount of Hsp70B/B′ mRNA was 4–5-fold higher in ED-LCLs than in primary B cells stimulated to proliferate by CD40L/IL-4 (Fig. 5b[Fig f5]).

Similar co-IP experiments to those described in Fig. 4 were performed by using LCL extracts (Fig. 5c[Fig f5]). EBNA3A in ED-LCL co-immunoprecipitated with Hsp70B′, Hsp40 and Bag3, but not with Hsp90*α*. The observed interactions were weak, but were significantly above the background produced by a non-specific antibody and were reproduced in a second independent LCL (data not shown). Again, the interaction was specific for EBNA3A; EBNA3C failed to interact with any of the chaperones and co-chaperones in LCLs (data not shown). These results confirmed that EBNA3A interacts with specific chaperones and co-chaperones even when it is expressed at its physiologically functional level in EBV-immortalized B cells.

### EBNA3A induces Hsp70, Hsp70B′ and Hsp40 in an adenovirus-free inducible system and EBNA3A expression results in a cytoplasm to nucleus shift of Hsp70

**Fig. 6. f6:**
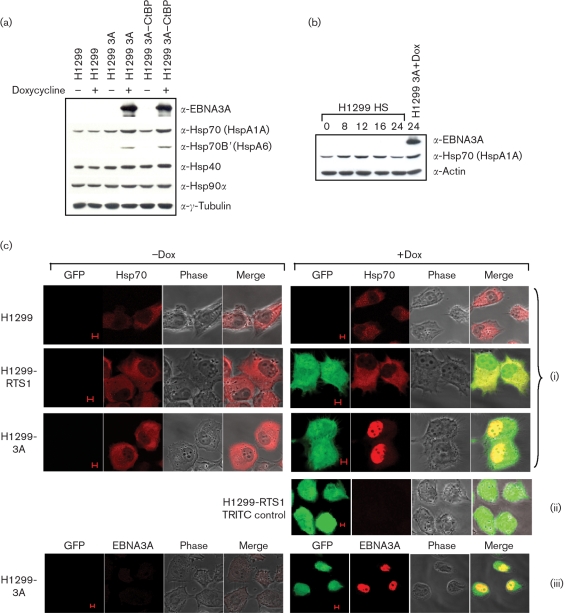
EBNA3A and the EBNA3A–CtBP binding mutant both upregulate Hsp70, Hsp70B′ and Hsp40 in an inducible expression system. EBNA3A expression results in the redistribution of Hsp70 from the cytoplasm to the nucleus. (a) Stable H1299 cell lines with doxycycline (Dox)-inducible EBNA3A or EBNA3A–CtBP binding mutants were induced with 1 μg doxycycline ml^−1^ and harvested 24 h post-induction. EBNA3A and EBNA3A–CtBP induce the expression of Hsp70, Hsp70B′ and Hsp40, but not of Hsp90*α*. Control H1299 cells treated with doxycycline show that the induction was not due to doxycycline alone. (b) Hsp70 levels following heat shock are similar to the levels induced by EBNA3A. H1299 cells were heat-shocked (HS) at 43 °C for 1 h and protein samples were taken at 0, 8, 12, 16 and 24 h following treatment. Hsp70 levels at 12 h post-heat shock are similar to the level of Hsp70 protein following EBNA3A induction in H1299 cells. (c) H1299, H1299-RTS1 and H1299-3A cells were stained for Hsp70 following incubation in the presence or absence of 1 μg doxycycline ml^−1^ (red; i). The bidirectional RTS1-inducible promoter expresses GFP in the presence of doxycycline (green; i, ii, iii). Only EBNA3A-expressing cells exhibited a relocation of Hsp70 from the cytoplasm to the nucleus following induction with doxycycline. (ii) Immunofluorescence was performed with the secondary TRITC antibody alone to show background non-specific staining. (iii) Control staining was also performed to show that GFP and EBNA3A are present in the same cells, consistent with activation of a bidirectional promoter (EBNA3A, red; GFP, green). Bars, 5 μm.

To exclude the possibility that an interaction between EBNA3A and adenovirus proteins produced the co-ordinated upregulation of chaperone/co-chaperone proteins, an inducible system was used. H1299 cells stably transfected with tetracycline-inducible EBNA3A or EBNA3A–CtBP binding mutant were induced with doxycycline and incubated for 24 h to allow efficient protein expression before subsequent Western blot analysis (Fig. 6a[Fig f6]). EBNA3A and the EBNA3A-CtBP binding mutant similarly activated Hsp70, Hsp70B′ and Hsp40, although Hsp40 upregulation in general was to a lower extent, due to higher endogenous protein levels. This activation was not due to the effects of doxycycline alone, as H1299 control cells, with and without the addition of doxycycline, showed no difference in chaperone/co-chaperone levels; nor was the upregulation due to the presence of the RTS-1 GFP-expressing plasmid backbone, as H1299-RTS1 control cells treated with doxycycline showed no difference in chaperone levels (data not shown). Importantly, the levels of heat-shock protein upregulation by EBNA3A are similar to the levels following heat shock for approximately 12 h (Fig. 6b[Fig f6]). If EBNA3A and the Hsp70 complex interact, this suggests that they must occupy the same cellular location. To test this, the distribution of Hsp70 with and without the induction of EBNA3A in H1299 cells was determined by immunofluorescence. EBNA3A expression resulted in the redistribution of Hsp70 from the cytoplasm to the nucleus (Fig. 6c[Fig f6]). Control cells consistently showed a predominantly cytoplasmic localization of Hsp70 in the absence of EBNA3A expression.

### EBNA3A and the CtBP binding mutant both activate transcription of an Hsp70B promoter

**Fig. 7. f7:**
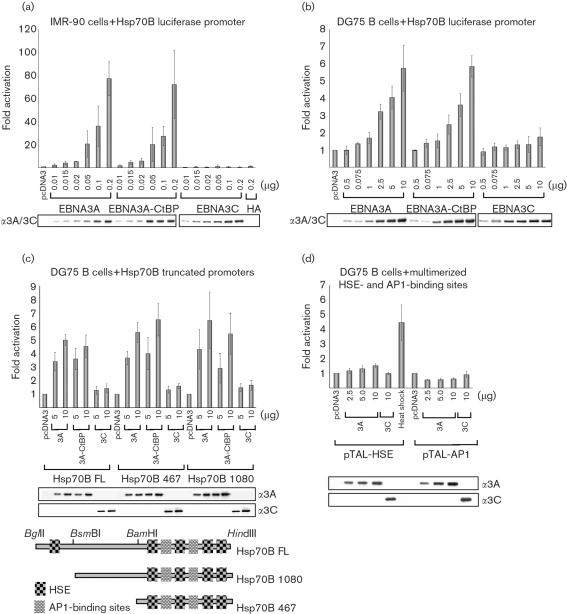
EBNA3A and the EBNA3A–CtBP binding mutant activate transcription from an Hsp70B promoter in IMR-90 fibroblasts and DG75 B cells. Truncations of the promoter highlight a 467 bp fragment through which EBNA3A can activate transcription. (a) IMR-90 cells were transfected with 40 ng pGL3-Hsp70B promoter luciferase reporter plasmid and pcDNA3-HA-EBNA3A, pcDNA3-HA-EBNA3A-CtBP binding mutant or pcDNA3-EBNA3C effector plasmid DNA as indicated. Cell extracts were prepared 48 h after transfection and luciferase activity was determined. Data were normalized to *β*-galactosidase activity (40 ng pSV-*β*gal per transfection was used) and are expressed as fold activation over pcDNA3 alone (which was given an arbitrary value of 1). Data from three independent experiments are shown, with sd indicated by error bars. (b) DG75 cells were transfected with 2 μg pGL3-Hsp70B luciferase reporter plasmid and effector plasmids as indicated. Means and sd from four independent experiments are shown. Data were normalized to *β*-galactosidase activity (2 μg pSV-*β*gal DNA per transfection was used). (c) DG75 cells were transfected as in (b), with two truncated Hsp70B promoter constructs. The truncated promoters and their respective regulatory elements are shown schematically. Means and sd from three independent experiments are shown. (d) EBNA3A has little or no effect on synthetic promoters containing either multimerized HSEs (pTAL-HSE, which was activated by heat shock for 1 h at 43 °C 24 h post-transfection) or AP1-binding sites (pTAL-AP1). Transfections were performed in DG75 B cells and the data from three independent experiments, each including duplicate transfections, are shown. Western blot analysis of one representative experiment is shown for each transfection experiment, indicating that the level of protein expression was comparable for each expression vector.

In order to determine whether EBNA3A activates a heat shock-responsive promoter directly, transient transfections and luciferase assays were performed. These experiments were conducted initially in IMR-90 cells using an Hsp70B luciferase promoter (pGL3-Hsp70B) together with EBNA3A, EBNA3A–CtBP binding mutant or EBNA3C expression vectors (Fig. 7a[Fig f7]). Transfection with increasing amounts of EBNA3A plasmid DNA (and the CtBP binding mutant) activated the Hsp70B promoter up to 70–80-fold more than transfection with empty vector alone. In contrast, EBNA3C failed to activate Hsp70B when similar amounts of plasmid were used.

Similar transfections were performed in DG75 Burkitt's lymphoma-derived B cells. The results shown in Fig. 7(b) revealed that both EBNA3A and the CtBP binding mutant activated Hsp70B by approximately 6-fold with 10 μg DNA. This was in contrast to EBNA3C, which failed to activate transcription of Hsp70B. DG75 cells were observed to have a higher background of Hsp70B promoter activity compared with IMR-90 cells, which have a very low basal activity. High basal activity of the heat-shock genes is common in grossly transformed cell lines (Calderwood *et al.*, 2006) and may in part explain the observed reduced activation when the general level of EBNA3A activation of Hsp70B is compared between DG75 and IMR-90 cells. It should be noted that although HA-tagged EBNA3A was used in all of the transient transfection experiments shown here, similar experiments using an untagged EBNA3A expression vector produced the same level of activation (data not shown).

These results suggest that EBNA3A can upregulate Hsp70B through transcriptional activation and that the loss of the ability of EBNA3A to bind CtBP has no effect on promoter activation. Activation is specific for EBNA3A, as EBNA3C has no effect on the Hsp70B promoter.

### EBNA3A (and the EBNA3A–CtBP binding mutant) can activate Hsp70B transcription from a truncated Hsp70B promoter, but not from multimerized heat-shock elements (HSEs) or AP1-binding sites

Hsp70B promoter sequence analysis and transcription factor-binding site prediction (using a promoter extending 1318 bp upstream of the transcription start site and transcription binding-site analysis tools available at http://www.cbrc.jp/research/db/TFSEARCH.html) revealed a number of HSEs (Fig. 7c[Fig f7]). Heat shock results in the activation of heat-shock factors (HSFs), which, through a process of trimerization and nuclear translocation/localization, results in the transcriptional activation of heat-shock genes by binding to HSEs (reviewed by Morimoto, 1998). To determine the minimal promoter region required for EBNA3A-mediated activation of Hsp70B, promoter truncations were produced (Fig. 7c[Fig f7]). Truncation to a 1080 bp promoter (removing the 5′ distal HSE) and further truncation to a 467 bp fragment both resulted in a response similar to that of the full-length Hsp70B promoter. The remaining promoter element contained four HSE sites and, in addition, two predicted AP1-binding sites (Fig. 7c[Fig f7]). Luciferase reporter assays using promoter elements with either multimerized HSEs or AP1-binding sites were performed; however, neither was activated significantly following co-transfection with EBNA3A (Fig. 7d[Fig f7]). These data suggest a mechanism for Hsp70B activation that is independent of HSF activation of HSEs and of the binding of factors to AP1 sites.

## DISCUSSION

Our studies using recombinant adenoviruses have demonstrated that the EBV oncoprotein EBNA3A can specifically coordinate the induction of Hsp70 chaperones and a specific subset of associated co-chaperones. Adenovirus expressing the closely related EBNA3C consistently failed to induce any of the chaperones or co-chaperones tested. Furthermore, the induction by EBNA3A is independent of any synergy between EBNA3A and adenovirus-encoded proteins, as activating EBNA3A expression in a stable, inducible system produced similar results.

By using Hsp70B as a representative heat-shock stress-inducible promoter, EBNA3A (but not EBNA3C) can activate transcription. Hsp70B-truncation experiments highlighted a minimal promoter region through which transcription activation was induced by EBNA3A. This promoter region contained a number of HSEs and AP1-binding sites. However, artificial promoters containing either multimerized HSEs or AP1-binding sites were not activated by EBNA3A, ruling out promoter activation by a simple, generic HSF mechanism. This suggests that EBNA3A activation was either independent of the HSE/HSF-binding sequences or that the precise promoter context (spacing and number of sites) is essential in the observed response. This is consistent with the specificity of the chaperones and co-chaperones that are induced by EBNA3A. Although Hsp70B is activated by HSF1 through HSE binding (He *et al.*, 2003; Stanhill *et al.*, 2006), the lack of a generic HSF-mediated promoter upregulation is consistent with the lack of a ‘blanket’ heat-shock protein response to EBNA3A expression. For example, microarray analysis showed no induction of related heat-shock genes, such as Hsp60 or Hsp105, which are also regulated by HSF1 in mammalian cells (Trinklein *et al.*, 2004; Supplementary Table S1, available in JGV Online).

EBNA3A, like the other EBNA3s, has no demonstrable DNA-binding ability, but has been shown to associate with DNA through the cellular DNA-binding adaptor protein RBP-J*κ* (Cludts & Farrell, 1998). However, the minimal Hsp70B promoter is devoid of any RBP-J*κ*-binding sites. There is also no evidence that the association of EBNA3A with CtBP (Hickabottom *et al.*, 2002) contributed to the observed induction of Hsp70B transcription, as both the wild-type EBNA3A and the CtBP binding mutant activated transcription to similar levels.

Co-IP experiments revealed that EBNA3A not only upregulates, but also interacts with, Hsp70/70B′, Hsp40 and Bag3. From IP of Hsp40, we have found that both EBNA3A and Hsp70 are co-precipitated following Western blot with respective antibodies. The interaction of Hsp40 with Hsp70 has been established previously (Demand *et al.*, 1998) and the results suggest the possibility of a ternary Hsp70–Hsp40–EBNA3A complex. It has been determined previously that both Hsp40 and Bag3 can bind Hsp70 proteins at distinct N- and C-terminal binding domains, respectively (Demand *et al.*, 1998; Doong *et al.*, 2003; Takayama *et al.*, 1999). Given that EBNA3A also binds Bag3, there is the potential for EBNA3A to nucleate/participate in a chaperone–co-chaperone complex.

Consistent with the hypothesis that EBNA3A forms a complex with Hsp70 and partners following EBNA3A expression in a stable, inducible cell line, Hsp70 redistributes from the cytoplasm to the nucleus (Fig. 6c[Fig f6]). The shift in chaperone localization to the site of EBNA3A function provides compelling evidence that EBNA3A and Hsp70 interact and suggests that it is functionally important. The interaction may not be linked exclusively to protein folding; Hsp70 chaperones are also associated with proteasome-mediated degradation, apoptosis and antigen presentation (Beere & Green, 2001; Esser *et al.*, 2004; Mosser & Morimoto, 2004; Srivastava, 2002). At this stage, we do not know which, if any, of these activities relate to EBNA3A expression.

The predicted secondary structure of EBNA3A suggests that the protein contains large regions of unstructured (natively unfolded) polypeptide (Touitou *et al.*, 2005). *In vitro* degradation assays have shown that EBNA3A is degraded readily by 20S proteasomes, but conversely, in latently infected B cells, EBNA3A is very stable with little *de novo* protein synthesis (Touitou *et al.*, 2005). After primary infection of resting B cells and the establishment of LCLs, the level of Hsp70 is seen to increase (Fig. 5). The interaction of EBNA3A with the Hsp70 chaperone complex may act to ensure correct protein conformation of newly synthesized proteins and as such protect EBNA3A from proteasome degradation. This is consistent with Bag3, but not Bag1, upregulation following EBNA3A expression (Fig. 2d[Fig f2]). Bag3 can abrogate Hsp70 complex and proteasome-mediated degradation by acting after polyubiquitination has occurred (Doong *et al.*, 2003). Hsp70 chaperone complexes that assist proteasome-mediated degradation typically form complexes containing Bag1 and ubiquitin ligase proteins such as CHIP (reviewed by Esser *et al.*, 2004). EBNA3A protein folding may be assisted through further interactions with chaperonins. EBNA3A has previously been shown to bind to the ε subunit of the cytosolic chaperonin TCP-1 ring complex (TRiC, also known as CCT; Kashuba *et al.*, 1999). It is unlikely, however, that all EBNA3A associates with Hsp70 complexes or that all Hsp70 complexes are protective. Hsp70 complexes have been linked with activation of antigen-presenting cells, cross-priming of antigen and processing of antigen for presentation by the major histocompatibility complex (reviewed by Srivastava, 2002), which may be important for the prevention of EBV-driven disease and maintenance of an asymptomatic infection.

EBV-positive Burkitt's lymphoma-derived cells are relatively resistant to cytotoxic drugs such as nocodazole, taxol and ionomycin, and it has been suggested that the EBNA3s may be involved in this phenotype (Kelly *et al.*, 2005; Leao *et al.*, 2007). Bag family proteins, in addition to modulating Hsp70 proteins, have been shown to work in synergy with Bcl2 in overcoming Bax-induced and FasL/Fas-mediated apoptosis (Antoku *et al.*, 2001; Lee *et al.*, 1999; Takayama *et al.*, 1995). Overexpression of Hsp70 alone can also prevent stress-induced apoptotic death through a mechanism that may require its chaperone function (Gabai *et al.*, 1997; Mosser *et al.*, 1997, 2000). Hsp70 has been shown to affect apoptotic signalling regulation, effector-molecule activation and events downstream of caspase activation (reviewed by Beere & Green, 2001; Mosser & Morimoto, 2004). Although the precise mechanism by which EBV enhances the survival of B cells exposed to cytotoxic drugs is unknown, and other viral factors could be responsible for the observed phenotype, EBNA3A upregulation of Hsp70 and Bag3 could contribute to the observed reduction in programmed cell death.

In summary, EBNA3A can specifically coordinate the upregulation of a distinct subset of chaperones and co-chaperones, and EBNA3A expression results in relocalization of Hsp70 to the nucleus. EBNA3A activates transcription of an Hsp70B promoter, but not of a promoter containing multimerized HSEs, suggesting greater specificity than a generic HSE/HSF response. Association of EBNA3A with this subset of chaperones and co-chaperones could ensure protein stability and may contribute to an EBV-mediated anti-apoptotic phenotype and/or antigen presentation.

## Supplementary Material

[Supplementary methods and tables]

## Figures and Tables

**Table 1. t1:** Chaperone and co-chaperone subset of normalized microarray data Values are fold induction of RNA in Ad-3A-infected IMR-90 cells compared with cells infected with the control empty adenovirus, Ad-E. All adenovirus infections were at an m.o.i. of 25 for 24 h before harvesting. Microarrays were performed with Sanger Hver2.1.1, containing 15 000 spotted human cDNA probes. All values are results of three independent experiments with six chips in total per experiment (with an incorporated dye swap). Microarray data have been Lowess-normalized.

Probe*	**Ad-3A vs Ad-E**		
	**Normalized**	**sd**	***t*-test *P* value**
Hsp70 (HspA1A)	2.202	0.784	5.44×10^−6^
Hsp70B/B′ (HspA6/7)	5.933	2.528	2.28×10^−7^
	2.623	1.102	8.86×10^−6^
Bag3	3.470	1.935	4.63×10^−5^
	3.221	1.273	1.04×10^−6^
Hsp40 (DNAJA1)	2.061	0.810	3.49×10^−5^

*There are two probes corresponding to Hsp70B′ and Bag3, but only one probe for Hsp70 (HspA1A) and Hsp40 (DNAJA1), because of redundancy of some probes on Sanger Hver2.1.1 chips.
