# Supplementary material for: Epstein–Barr virus nuclear antigen (EBNA) 3A induces the expression of and interacts with a subset of chaperones and co-chaperones
Source: J Gen Virol. 2008 Apr;89(Pt 4):866–77. doi: 10.1099/vir.0.83414-0 (PMC2885026; doi:10.1099/vir.0.83414-0)
Supplement: [Supplementary methods and tables] [file supp_89_4_866__index.html]

 Epstein-Barr virus nuclear antigen (EBNA) 3A induces the expression of and interacts with a subset of chaperones and co-chaperones -- Young et al. 89 (4): 866 Data Supplement - Supplementary methods and tables -- Journal of General Virology

### Epstein–Barr virus nuclear antigen (EBNA) 3A induces the expression of and interacts with a subset of chaperones and co-chaperones, by P. Young, E. Anderton, K. Paschos, R. White and M. J. Allday

*Journal of General Virology* vol. **89**, part 4, pp. 866 – 877

**Supplementary Methods.** Adenovirus construction, transfection methods for reporter assays and inducible EBNA3A cloning

**Supplementary Tables S1 and S2.** Normalized full microarray tables, showing all genes upregulated or downregulated as a consequence of comparing Ad-3A with Ad-E IMR-90 infection

[Single PDF file] (328 KB)

  
  
