# Supplementary material for: Epstein–Barr virus nuclear antigen (EBNA) 3A induces the expression of and interacts with a subset of chaperones and co-chaperones
Source: J Gen Virol. 2008 Apr;89(Pt 4):866–77. doi: 10.1099/vir.0.83414-0 (PMC2885026; doi:10.1099/vir.0.83414-0)
Supplement: [Supplementary methods and tables] [file supp_89_4_866__1.pdf]

SUPPLEMENTARY METHODS

Adenovirus construction.

EBNA3A and the 3A-CtBP binding mutant were cloned from pcDNA3-HA-EBNA3A and pcDNA3-HA-EBNA3A-CtBP, respectively, by restriction digest with *Bam*HI (partial digestion) and *Eco*RV. The resulting EBNA3A fragment was ligated into p-Shuttle-CMV cut with the same combination of restriction enzymes. EBNA3C was cloned initially into pBluescript II SK+, forming a cloning intermediate to give the appropriate restriction sites to allow further cloning into p-Shuttle-CMV. EBNA3C was initially cloned from a pcDNA3-EBNA3C vector by restriction digest with *Hin*dIII and *Not*I and ligated into pBluescript II SK+ digested with the same combination of restriction enzymes. The resultant pBluscript-EBNA3C plasmid was digested with *Sal*I, *Not*I and *Bss*HII for ligation. *Bss*HII prevented reannealing of EBNA3C and pBluescript on ligation with p-Shuttle-CMV (digested with *Sal*I and *Not*I).

Cloned Shuttle vectors were linearized with *Pme*I and electroporated into *recA*$^+$ *Escherichia coli* BJ5183 cells (50 µl, 2500 V, 200 Ω and 25 µF in a Bio-Rad Gene Pulser electroporator with 2.0 mm cuvettes) stably expressing the pAdEasy-1 adenoviral backbone plasmid. Resultant recombinant colonies were diagnosed by restriction digest with *Pac*I or *Hin*dIII.

Adenovirus vector construct (1 µg) was linearized with *Pac*I, ethanol-precipitated and transfected [FuGENE 6, as per the manufacturer's instructions (Roche)] into 4×10^5 293A packaging cells per well of a six-well plate. Ten to 14 days post-transfection, when 70–80% of cells showed adenoviral cytopathic effect (Ad CPE), cells were harvested. Virus was released from the cells by three freeze–thaw steps with vortexing between each cycle. Half of the virus suspension was used to infect an increasing number of cells. First, 10 cm dishes were seeded at a density of 2×10^6 and infected with the virus suspension. Once cells showed considerable CPE, cells were harvested and virus released by freeze–thaw. Finally, a 175 cm^2 flask was seeded at a density of 5.5×10^6 and infected with virus suspension.

For large-scale adenoviral vector production, a ten-chamber cell factory (CF-10; NUNC) was seeded with 293A cells and infected with half of the adenovirus-infected cells from the 175 cm^2 flask until the cells showed visible signs of detachment. On detachment, cells were harvested and washed in cold PBS before being resuspended in 12 ml cold 10 mM Tris/HCl, pH 8.0, and frozen and thawed three times with vortexing between each cycle. Cell debris was pelleted by centrifugation at 4300 ***g*** for 10 min at 4 °C, and the virus-containing supernatant was collected and incubated on ice.

Young, P., Anderton, E., Paschos, K., White, R. & Allday, M. J. (2008). Epstein–Barr virus nuclear antigen (EBNA) 3A induces the expression of and interacts with a subset of chaperones and co-chaperones. *J Gen Virol* **89**, 866–877.

Purification of the adenovirus vector was by caesium chloride (CsCl) gradient. A CsCl gradient was prepared by layering 11.4 ml of 1.25 g CsCl ml^{-1} [36.16 g CsCl in 100 ml TD solution (25 mM Tris/HCl buffer, pH 7.5, containing 136 mM NaCl, 5 mM KCl, 0.7 mM NaH_2PO_4)] over 7.6 ml of 1.4 g CsCl ml^{-1} (62 g CsCl in 100 ml TD solution) in a 36 ml ultraclear centrifuge tube (Beckman). The viral lysate was layered onto the gradient and centrifuged at 25 000 r.p.m., using a SW28 Beckman swing-out rotor, for 2 h at 15 °C in a Sorvall Combi ultracentrifuge. After centrifugation, the virus band was positioned between the gradients beneath the cellular debris. A band of empty capsids is positioned above the band of viable particles. The viable virus was extracted by puncturing the side of the centrifuge tube below the band with a 19-gauge needle. The extracted virus was placed in a 5 ml ultraclear centrifuge tube (Beckman), overlaid to the top with 1.35 g CsCl ml^{-1} (51.2 g CsCl in 100 ml TD solution) and centrifuged for 16 h at 40 000 r.p.m. at 15 °C in an SW55Ti Beckman swing-out rotor. The band of viable particles was extracted as described above.

The CsCl virus solution was diluted in 1 vol filter-sterilized 60% sucrose and desalted by dialysis. Dialysis was conducted by using a 3500 MWCO Slide-a-lyser dialysis cassette (Perbio) in 1 l dialysis buffer (10 mM Tris, pH 8.0, 1 mM $MgCl_2$, 150 mM NaCl, 50 mg Tween 80 l^{-1} and 1 M sucrose) with stirring at 4 °C. The dialysis buffer was changed three times with two 3 h dialysis steps and one prolonged overnight dialysis. The virus was aliquotted and stored at –80 °C. The CsCl-purified adenoviral stocks were titrated by using a BD Clontech Adeno-X-rapid titre kit (as per the manufacturer's instructions).

Transfection methods for reporter assays.

For DG75 reporter assays, 2 μg SVβ-gal, 2 μg promoter (pGL3-Hsp70B, pGL3-Hsp70B 1080, pGL3-Hsp70B 467, pHSE-luc or pAP1-luc) and varying amounts of pcDNA3-HA-EBNA3A or pcDNA3-EBNA3C were used. pGL3-Hsp70B 1080 promoter element was constructed by a *Bgl*II–*Bsm*BI blunt ligation of pGL3-Hsp70B plasmid. pGL3-Hsp70B 467 was constructed by digestion of pGL3-Hsp70B with *Bgl*II–*Xba*I and *Bam*HI–*Xba*I, followed by religation of the *Xba*I sites and ligation of *Bgl*II to *Bam*HI. The total amount of DNA per transfection was balanced with the same amount of control vector DNA. A standard number of DG75 cells (1×10^7) were electroporated per transfection. Electroporation was conducted at 250 V, 960 μFa, using a Gene Pulser (BioRad). Cells were incubated at 37 °C for 48 h before harvesting.

IMR-90 cells were seeded at 1.5×10^5 per well in a six-well plate 24 h before transfection. The LID transfection system, consisting of Lipofectin reagent (Invitrogen), integrin-targeting peptide 6 (Hart *et al.*, 1998; White *et al.*, 2003) and DNA, was used. For 1 μg plasmid DNA, 0.75 μl Lipofectin reagent (1 mg ml^{-1}) was combined with 40 μl peptide 6

(0.1 mg ml^{-1}) and left to stand for 5 min. For transient IMR-90 transfections, 0.28 µg plasmid DNA was used, with 40 ng promoter and 40 ng SVβ-gal. DNA was made up to 100 µl in OptiMEM (Invitrogen) and combined with the peptide and Lipofectin mix and allowed to stand for 20 min. The DNA–peptide 6–Lipofectin complex was increased in volume to 1 ml with OptiMEM and added to cells. Cells were left for 6 h before washing and subsequently incubated for 48 h post-transfection before harvesting.

Inducible EBNA3A cloning.

Briefly, HA-EBNA3A and HA-EBNA3A-CtBP were excised from pcDNA3-HA-EBNA3A and pcDNA3-HA-EBNA3A-CtBP (Hickabottom *et al.*, 2002) with *Hin*dIII/*Not*I digestion and cloned into pUC19-*Sfi*I. They were then excised from pUC19-*Sfi*I after digestion with *Sfi*I and cloned into pRTS1-SVP [pRTS1 as described by Bornkamm *et al.* (2005)].

Supplementary Tables S1 and S2. Normalized full microarray tables, showing all genes upregulated (Supplementary Table S1) or downregulated (Supplementary Table S2) as a consequence of comparing Ad-3A with Ad-E IMR-90 infection

All adenovirus infections were at an m.o.i. of 25 for 24 h before harvesting. Microarrays were performed with Sanger Hver2.1.1, containing 15 000 human spotted cDNA probes; the full list of upregulated or downregulated genes is presented following Lowess normalization. The table shows 12 genes that have been 2-fold upregulated following EBNA3A expression. Microarray analysis was not able to distinguish between several Hsp70 genes; HspA1A, 1B or 1L and HspA6 or HspA7. This is due to high sequence similarity. Nucleotide sequence analysis of the two intron-less genes HspA1A and HspA1B has shown that they are almost identical and encode an identical protein product [and hence microarrays are unable to distinguish due to the potential for cross-hybridization; Milner & Campbell (1990)]. HspA1L has 90 % amino acid sequence similarity to HspA1A, but is expressed constitutively at low levels and, unlike HspA1A and 1B, is not inducible (Milner & Campbell, 1990). HspA1L has not been analysed further. Similarly, Hsp70B and B′ (HspA6 and HspA7) have 98 % nucleotide identity and thus microarrays are unable to distinguish between the mRNAs (Parsian *et al.*, 2000). Despite the high nucleotide identity, Hsp70B mRNA does not encode a functional protein, due to a premature stop codon (Parsian *et al.*, 2000). HspA2 is expressed constitutively at low levels in most tissues, but is high in testis and skeletal muscle (Bonnycastle *et al.*, 1994). HspA2 was not analysed further. Microarray analysis also showed upregulation of Hsp90α. However, Western blot analysis showed no induction of Hsp90α protein.

Supplementary Table S1

Sanger Accession No.	Fold Change	t-test P-value	Gene Name	Description
33800_A	5.93	2.28E-07	HSPA6;HSPA7	Heat shock 70 kDa protein 6 (Heat shock 70 kDa protein B')
8425_1	4.02	3.84E-10		No Annotation
stSG493479	3.47	4.63E-05	BAG3	BAG-family molecular chaperone regulator-3 (BCL-2 binding athanogene- 3) (BAG-3) (Bcl-2-binding protein Bis) (Docking protein CAIR-1)
stSG416190	3.22	1.04E-06	BAG3	BAG-family molecular chaperone regulator-3 (BCL-2 binding athanogene- 3) (BAG-3) (Bcl-2-binding protein Bis) (Docking protein CAIR-1).
stSG415320	2.62	8.86E-06	HSPA6;HSPA7	Heat shock 70 kDa protein 6 (Heat shock 70 kDa protein B').
1844_1	2.36	5.32E-05	HSPA2	Heat shock-related 70 kDa protein 2 (Heat shock 70 kDa protein 2).
stSG443658	2.26	2.64E-06	CDCP1	CUB domain-containing protein 1 isoform 1 [Homo sapiens].
15597_1	2.20	5.44E-06	HSPA1A;HSPA1B;HSPA1L	Heat shock 70 kDa protein.
222155_B	2.17	2.86E-05	HSPCA	Heat shock protein HSP 90-alpha (HSP 86).
stSG494069	2.12	9.54E-11	SPHK1	Sphingosine kinase 1 (EC 2.7.1.-) (SK 1) (SPK 1).
44394_B	2.06	3.49E-05	DNAJA1	DnaJ homolog subfamily A member 1 (Heat shock 40 kDa protein 4) (DnaJ protein homolog 2) (HSJ-2) (HSDJ).
stSG415919	2.00	1.43E-07	ITPR3	Inositol 1,4,5-trisphosphate receptor type 3 (Type 3 inositol 1,4,5- trisphosphate receptor) (Type 3 InsP3 receptor) (IP3 receptor isoform 3) (InsP3R3).

Supplementary Table S2

Sanger Accession No.	Fold Change	t-test P-value	Gene Name	Description
stSG489769	-2.08	6.28E-10	CCL2	Small inducible cytokine A2 precursor (CCL2) (Monocyte chemotactic protein 1) (MCP-1) (Monocyte chemotactic and activating factor) (MCAF)
stSG444220	-1.91	3.44E-10		MSTP014 - Hypothetical protein

REFERENCES

Bonnycastle, L. L., Yu, C. E., Hunt, C. R., Trask, B. J., Clancy, K. P., Weber, J. L., Patterson, D. & Schellenberg, G. D. (1994). Cloning, sequencing, and mapping of the human chromosome 14 heat-shock protein gene (HSPA2). *Genomics* **23**, 85–93. Medline

Bornkamm, G. W., Berens, C., Kuklik-Roos, C., Bechet, J. M., Laux, G., Bachl, J., Korndoerfer, M., Schlee, M., Holzel, M. & other authors (2005). Stringent doxycycline-dependent control of gene activities using an episomal one-vector system. *Nucleic Acids Res* **33**, e137. Medline

Hart, S. L., Arancibia-Carcamo, C. V., Wolfert, M. A., Mailhos, C., O'Reilly, N. J., Ali, R. R., Coutelle, C., George, A. J., Harbottle, R. P. & other authors (1998). Lipid-mediated enhancement of transfection by a nonviral integrin-targeting vector. *Hum Gene Ther* **9**, 575–585. Medline

Hickabottom, M., Parker, G. A., Freemont, P., Crook, T. & Allday, M. J. (2002). Two nonconsensus sites in the Epstein–Barr virus oncoprotein EBNA3A cooperate to bind the co-repressor carboxyl-terminal-binding protein (CtBP). *J Biol Chem* **277**, 47197–47204. Medline

Milner, C. M. & Campbell, R. D. (1990). Structure and expression of the three MHC-linked HSP70 genes. *Immunogenetics* **32**, 242–251. Medline

Parsian, A. J., Sheren, J. E., Tao, T. Y., Goswami, P. C., Malyapa, R., Van Rheeden, R., Watson, M. S. & Hunt, C. R. (2000). The human Hsp70B gene at the HSPA7 locus of chromosome 1 is transcribed but non-functional. *Biochim Biophys Acta* **1494**, 201–205. Medline

White, R. E., Wade-Martins, R., Hart, S. L., Frampton, J., Huey, B., Desai-Mehta, A., Cerosaletti, K. M., Concannon, P. & James, M. R. (2003). Functional delivery of large genomic DNA to human cells with a peptide-lipid vector. *J Gene Med* **5**, 883–892. Medline

---

Young, P., Anderton, E., Paschos, K., White, R. & Allday, M. J. (2008). Epstein–Barr virus nuclear antigen (EBNA) 3A induces the expression of and interacts with a subset of chaperones and co-chaperones. *J Gen Virol* **89**, 866–877.
